# Infusing wellness opportunities into integrated youth services

**DOI:** 10.1186/s12888-023-04809-6

**Published:** 2023-06-05

**Authors:** Krista Glowacki, Jennifer Affolder, Brooke Macnab, Alayna Ewert, Karen Tee, Matt Wenger, Godwin Chan, Steve Mathias, Skye Barbic

**Affiliations:** 1 Faculty of Medicine, Department of Occupational Science and Occupational Therapy, The University of British Columbia, T325 – 2211 Wesbrook Mall, Vancouver, BC V6T 2B5 Canada; 2 Centre for Health Evaluation & Outcome Sciences, Providence Health Care, #588-1081 Burrard Street, Vancouver, BC V6Z 1Y6 Canada; 3 Foundry, 915-1045 Howe Street, Vancouver, BC V6Z 2A9 Canada; 4 Therapeutic Recreation, Douglas College, PO Box 2503, New Westminster, BC V3L 5B2 Canada; 5 Providence Research, 10th Floor - 1190 Hornby Street, Vancouver, BC V6Z 2K5 Canada

**Keywords:** Wellness, Youth, Mental health, Substance use, Integrated youth services, Health promotion, Leisure activities

## Abstract

**Background:**

Appropriate health services and health promotion strategies for young people with mental health and substance use (MHSU) concerns are critical for recovery. Foundry, an integrated youth services (IYS) initiative for young people ages 12-24 in British Columbia (BC), Canada, has recently added leisure and recreational activities (referred to as the Wellness Program) into its services. The objectives of this study were to: (1) describe how the Wellness Program was implemented over a two-year period into IYS (2) provide an overview of what the Wellness Program is, who accessed the program since inception and initial evaluation results.

**Methods:**

This study was part of the developmental evaluation of Foundry. A phased approach was used to implement the program at nine centres. Data was accessed from Foundry’s centralized platform ‘Toolbox’ and included activity type, number of unique youth and visits, additional services sought, information about how youth found out about the centre, and demographics. Qualitative data was also accessed from focus groups (*n*=2) conducted with young people (*n*=9).

**Results:**

Over the two-year period, 355 unique youth accessed the Wellness Program, with 1319 unique visits. Almost half (40%) of youth identified the Wellness Program as the first point of access to Foundry. A total of 384 different programs were offered targeting five wellness domains (physical, mental/emotional, social, spiritual, and cognitive/intellectual). The majority of youth identified as young girls/women (58.2%), 22.6% as gender diverse, and 19.2% as young men/boys. The mean age was 19 years, and most participants were between the ages of 19-24 years (43.6%). From the thematic analysis of focus groups, we found young people enjoyed the social aspect of the program with peers and facilitators, and identified program improvements that are being considered as the program grows.

**Conclusions:**

This study provides insight into the development and implementation of leisure-based activities (known as the Wellness Program) into IYS and can be used as a guide by international IYS initiatives. The initial reach of programs over two years is promising, and these programs are acting as a potential gateway for young people to access other health services.

**Supplementary Information:**

The online version contains supplementary material available at 10.1186/s12888-023-04809-6.

## Background

Mental health and substance use (MHSU) challenges present early in life, as 50-70% develop during childhood or adolescence [1], and the peak age of onset is 14.5 years [2]. In Canada, MHSU disorders affect 1 in 4 youth, with 12-24-year-old individuals experiencing the highest incidence of MHSU disorders of any age group [3, 4]. Population mental health concerns exist across all ages and have been exasperated by the COVID-19 pandemic, with some suggesting a global mental health crisis [5, 6]. Young people are a particularly at-risk population during this time with increasing mental health concerns [7, 8]. It has been suggested that young people have been disproportionately affected by the pandemic with employment loss, social isolation, and loss of infrastructures and supports such as school closures [9, 10]. Stemming from this, an international call has been made to invest in services to support the long-term mental health needs of young people, including health promotion/prevention, early intervention, and crisis care [6, 10]. As we navigate pandemic phases and recovery, it is vital now more than ever to intervene with the full range of MHSU services and interventions including health promotion strategies for young people.

Leisure-based activities (defined as voluntary and non-workplace or educational aspects of social life) have been highly debated throughout the COVID-19 pandemic [11]. Norman and colleagues [11] analyzed how leisure activities were understood and represented in media coverage during the pandemic in Canada. The authors acknowledge the contention about restrictions and closures perpetuating anger and frustrations, but also about leisure activities being a source of hope when re-opening and their potential for the health and economic recovery for Canadians. Leisure activities have the potential to play an important role for health promotion and prevention of worsening mental and physical health for young people. They can reduce stress, provide protective health benefits, play a role in recovery from mental illness [12], improve self worth and self-esteem [13], and promote quality of life [14]. Jae-Kim and Cho [15] identified the potential role leisure activities can play during the current pandemic and future pandemics in South Korea. A Finnish study identified that high social leisure time in adolescence was associated with a lower incidence of psychiatric disorders, particularly affective, anxiety, and substance use disorders [16]. Participation in leisure-based activities with peers has been shown to reduce feelings of social isolation and to promote psychological stability during the pandemic [15]. However, activities with peers need infection control and modification. For instance, physical activity (bodily movement that results in energy expenditure) can be modified to either do safely with peers (e.g., outdoors, virtually from home) or individually. Physical activity has been recommended as an important strategy to reduce the increased MHSU challenges brought on by the pandemic and lockdowns [17, 18], and acknowledged as a valuable health promotion tool for young people [19–21]. 

Integrated Youth Services (IYS) are community-based (preferably in a single location), youth- and family-centred, and provide multidisciplinary care in a youth-friendly environment [6, 22, 23]. The needs of young people and their families/caregivers are prioritized in the design of services and care [24]. IYS initiatives include headspace™ in Australia [25], Jigsaw™ in Ireland [26], Les Maisons des Adolescents in France [27], and Youth One Stop Shops (YOSS) in New Zealand [28]. Canadian IYS initiatives include Foundry [24, 29] and Youth Wellness Hubs Ontario (YWHO) [29]. IYS are a model of care that can also provide leisure-based activities as part of their services. Participation in leisure is identified as a goal for many youths accessing IYS [24], however little is known about the implementation of leisure-based activities for health promotion within this model. Leisure-based activities encompass and will be referred to as the Wellness Program for the purpose of this study. As part of developmental evaluation, the objectives of this study were to (1) describe how the Wellness Program was implemented and evaluated over a two-year period into IYS in British Columbia (BC), Canada, and (2) provide an overview of what the Wellness Program is,who accessed the program since inception, and initial program evaluation results.

## Methods

### Context

Foundry is an IYS that provides care for young people ages 12-24 years in BC, Canada [24] and as of May 2023, fifteen Foundry centres are open, with seven more in development. The centres exist in communities representative of small, medium, and large population centres in Canada [30]. Virtual services also support youth from across the province [8]. Foundry has five core service streams, including mental health, substance use, peer support, physical (including sexual) health, and social services. Based on an internal 2019 survey of 10,000 youth, Foundry identified a need to build leisure and physical activity into services not only as an adjunct to MHSU supports but in recognition that holistic care is central to individual wellness. With the support of private donors, Foundry implemented the Wellness Program at nine centres. Communities were asked to co-design programming that met the needs of youth accessing their centres.

### Study design

Ethics approval was received from the University of British Columbia (ID: H21-01510). This study was done as a component of Foundry’s developmental evaluation which has supported the implementation of Foundry’s complex, diverse, and innovative interventions and allowed the capacity for real time decision-making to change and adapt the evolving system [31, 32]. The program was designed and implemented over six phases (defined below) and evaluated using mixed methods primarily in Phase 5 and Phase 6. To support implementation, a multidisciplinary team of researchers, a research and evaluation associate, youth peer evaluators, and various leadership team members was created at Foundry Central Office (FCO), Foundry’s central administrative and implementation team.

### Program design and implementation

The following describes the phased approach used to design and implement the Wellness Program, and is a summary from a written log kept by the project coordinator during the timeline provided.

#### Phase 1: Community asset development September-October 2019

Communities were given the opportunity to assess the needs of youth in their region. The project coordinator informed centre staff at eight Foundry centres of the initiative to implement the Wellness Program. Staff then shared this by word of mouth with youth, family and peer support workers and those interested in providing input were given the project coordinator’s contact information. An environmental scan was conducted in collaboration with centre staff to identify assets, gaps and community need in services through team meetings, individual meetings, centre tours, discussions, and the administration of a survey (survey questions provided as supplementary material). The interested community members were asked to describe how meaningful activities – such as spending time outdoors, exploring creative expression, or participating in physical activities – could impact the wellness of diverse, Foundry-accessing youth. Examples of questions posed include, “What does Wellness mean to you? What does Wellness mean to the youth you serve? What community resources/services would you use? What are the main barriers for youth in accessing these resources/services?” The only restriction from the first funder was that programs needed to be offered in-person to reduce screen time. Proposals, budgets and requests for funding was reviewed with centre managers and teams and discussions were had about opportunities for community partnerships and resource sharing.

#### Phase 2: Gathering momentum September-December 2019

The project coordinator worked closely with eight centres and communities to support project management, access and flow funds, program co-development and ongoing engagement. The project coordinator liaised with a provincial dietician, and supported centre staff to identify assets and strengths in their communities. They also liaised with implementation coordinators at centres for collaborative work, and began exploring opportunities for youth empowerment with employment roles such as peer support workers or cultural coordinators, and explored Indigenous program opportunities. The project coordinator collaborated with FCO to: identify evaluation tools/protocols (e.g., integrating and tracking through Toolbox) and research opportunities, and implement group programming within IYS. Formal community partnerships were established with local and provincial agencies. Centres worked with their community members to develop and draft an outline for their Wellness Program and co-design a custom curriculum to meet their unique needs. The project coordinator sent initial draft curriculum content to centres. Centres then sought input from Youth Advisory Committees, peer support workers, and Indigenous youth and families. Content was also reviewed by FCO staff. Changes were then made to the content, specifically inclusion of the voice of youth in conceptualizing wellness, tools and templates for group development and facilitation, and risk management.

#### Phase 3: Pilot programs and partnerships January 2020-April 2020

Pilot Programs began at each centre (e.g., Healthy by Nature outdoor program). The project coordinator identified centre champions, developed waivers, liability and consent forms, as well as service agreements and contracts with community partner organizations. They also provided ongoing centre support and practical toolkits and resources while continuing to develop the wellness curriculum. In parallel, an evaluation team co-designed the evaluation framework and research questions to understand the impact of this pilot phase. Youth Peer Evaluators working with FCO’s Research and Evaluation teams were consulted when establishing a framework.

#### Phase 4: Pandemic pivot April 2020-December 2020

The COVID-19 global pandemic was declared in March 2020 [33]. Public health measures required communities to adapt programs to conform with provincial restrictions and regulations for in-person gatherings. To meet the coordination needs of communities, an FCO working group was formed to support implementation of the Wellness Program within the new pandemic restrictions. As it became apparent that the public health measures would last for an extensive period of time, several workshops were offered from FCO to support program facilitation. For instance, a group facilitation training session was offered for peer support workers across the centres. A need was identified to change the original curriculum into a user-friendly engaging practical resource (which became the Wellness Program Guide further described in Phase 6). The project coordinator explored definitions of wellness from the youth perspective at centres and from staff at Foundry Central Office.

#### Phase 5: Knowledge exchange and adaption January 2021-April 2021

The project coordinator continued to collaborate with community partners, provided a report and updates to funders, and held a Foundry network-wide knowledge exchange workshop to share information about the Wellness Program offered at different centres. They also supported centres with creative problem-solving during pandemic restrictions to offer hybrid program ideas or smaller in-person scenarios and began exploring opportunities for youth peer engagement within the Wellness Program and development of the program with an Indigenous perspective. A team comprised of a researcher, a research and evaluation associate, and four youth peer evaluators began program evaluation (including conducting of focus groups with young people).

#### Phase 6: Wellness program guide and evaluation April 2021-December 2021

The project coordinator consulted and worked with the Foundry Indigenous Wellness Team, centre champions and FCO team members to develop and publish the Wellness Program Guide which outlines program vision, intent, examples and working templates to run programs [34]. The Table of Contents of the Wellness Program Guide is included in Table 1 for reference. The aim established is the Wellness Program should complement other health services offered and target five domains of wellness: physical, social, emotional/mental, cognitive/intellectual, spiritual/cultural (See Table 2 for definitions) [35]. The vision statement is “Move your body, Calm your mind” [34]. Program objectives include the emphasis on social inclusion and connection with self, others, nature, engagement with community partners, and the enhancement of the service model by infusing wellness opportunities. Ongoing support was provided for centres to provide programming with constantly changing provincial restrictions.

**Table 1:** Table of contents of the wellness guide [34]

**Section**	**Section content**	**Pages**
Acknowledgements		1
Introduction	Background and Context of Wellness Program	2-4
	Purpose of This Guide	
What is Wellness?	Youth Perspective	6-12
	Accessible Wellness	
	Domains of Wellness	
	Indigenous Wellness	
	Tools and Resources	
Developing a Wellness Program at Your Centre	Assess Participant Needs and Community Assets	13-41
	Develop a Proposal	
	Plan and Design Program	
	Engage in Pre-Program Tasks	
	Implement Program	
	Evaluate Program	
Types of Wellness Activities	Community-Based Activities	45-49
	Nature-Based and Land-Based Activities	
	Small Group Activities	
	Large Group Activities	
	Individual Activities	
	Virtual Activities	
	Tools and Resources	
Activities Within the Domains of Wellness	Physical Wellness	50-62
	Emotional/Mental Wellness	
	Social Wellness	
	Cognitive/Intellectual Wellness	
	Spiritual/Cultural Wellness	
Community Partnerships	BC Parks Foundation	65-67
	Power To Be	
	Women’s National Field Hockey Team	
	YMCA	
	Creating New Partnerships	
Research	Wellness Research Projects	68-69
	Digital Storytelling as a Research Technique	
	Partnerships	
	Tools and Resources	
Sustaining Wellness		70
Tools and Resources		71-72
Contact List		73
Contributors		74
References		75

**Table 2:** Wellness domains and definitions adapted from Payne, Ainsworth, and Godbey [35]

**Wellness domain**	**Definition**	**Activity aim**
Physical	Move more, eat well, sleep better	Physical activity, nutrition, sleep, connections to nature, outdoor recreation
Social	Build connections, share experiences, find role models	Making friends and feeling included, sharing lived experiences, connecting with mentors and Elders, low-pressure activities, healthy relationship skills
Emotional/Mental	Express yourself, live in the moment, cope with stress	Stress management, self-care, relaxation and mindfulness, self-awareness and self-acceptance, hope and optimism
Cognitive/Intellectual	Boost your brain, try new things, empower yourself to lead	Creative and brain-boosting activities, leadership and volunteering opportunities, goal setting, exploring interests, learning new skills
Spiritual/Cultural	Feed your soul, find purpose, connect with your culture	Discovering purpose in life, spiritual practices, cultural activities, cultural teachings, creating harmony and connection with the earth

### Data collection and analyses

Ethics approval was received from the University of British Columbia Behavioural Research Ethics Board (ID: H21-01510). Data for this study were collected from two sources, the first being Foundry’s centralized data platform called ‘Toolbox’ for all young people who accessed the Wellness Program over a two-year period (September 2019- September 2021). These data are routinely collected in accordance with the BC Freedom of Information and Protection of Privacy Act (FIPPA) and the BC Personal Information Protection Act (PIPA) for the purposes of service delivery, evaluation and research. Informed consent was received from youth prior to survey completion, and youth do not have to complete the survey in order to receive services (i.e., completion of the surveys is voluntary). Youth voluntarily consent to the data being used for research purposes by indicating they have read and understood the purpose of the survey and how data are collected, stored and reported. Data are de-identified, stored, accessed, and analyzed in a secure research environment. Data are not linked in any way back to personal identifiers. Specific to the Wellness Program, data was collected on activity type, number of unique youth and visits. The forms also collected information about the type of services sought in addition to the Wellness Program (physical health, sexual health, mental health, substance use, youth peer support or social service); information about how the youth found out about Foundry; and demographic data including identity, age, sexual orientation, ethnic/cultural background. Descriptive analyses were conducted calculating proportions (n/N, %) for all variables.

In addition to ‘Toolbox’ data, qualitative data collected as part of Foundry’s developmental evaluation was also accessed. Young people (aged 16-24 years) who participated in the Wellness Program at any Foundry centre across BC, Canada since program inception (September 2019) were eligible to participate. Young people were invited to participate and recruited through Foundry’s social media and through staff offering the program at centres and offered a $30 gift card for participation. Verbal consent was gained by young people at the time of the focus group. These data are routinely collected in accordance with the BC FIPPA and the BCPIPA for the purposes of service delivery, evaluation and research (as further described above). Semi-structured virtual focus groups (*n*=2) were conducted with young people (*n*=9 participants) via the platform Zoom© from April-September 2021. It was optional for participants to have their video camera on, and participants could use the chatroom feature and type versus answer verbally. Focus groups were conducted to meet evaluation objectives outlined in (Table 3), and explore the experiences of young people. The focus group guide is provided as Supplementary Material.

**Table 3:** Program evaluation objectives

Objective 1.	To understand the extent to which the program was implemented as intended
Objective 2.	To understand the extent to which the wellness activities have enhanced Foundry’s services
Objective 3.	To understand young people’s satisfaction and involvement with the program and to understand the extent to which participants report the program impacted their health and wellness
Objective 4.	To understand the extent to which awareness and understanding of how technology is impacting youth is increased
Objective 5.	To understand the extent to which the activities have resulted in opportunities for collaboration and partnership

All focus groups were audio-recorded and transcribed verbatim. All exact text from the chat feature of zoom was integrated into the transcripts at the appropriate times. To maintain confidentiality, participant numbers were assigned to each participant, and any identifying information was removed from transcripts. QSR International’s NVivo © 12 Software was used to facilitate qualitative data analysis. A blend of deductive and inductive analytical approach was used in a multi-step process to conduct a thematic analysis [36]. This included becoming familiar with data- audio recordings were listened to, then re-listened to while reading and re-reading transcripts and notes were taken. Next, open broad codes were generated in NVivo© software based on the evaluation objectives and interview guide topics. Researcher (author KG) coded half of one focus group transcript with two youth peer evaluators to discuss coding, answer any questions and ensure understanding of the research process. The broader codes were then reviewed to search for and identify data-driven ideas and patterns (and ultimately developed into themes) and this step was considered inductive. Themes were then reviewed across and within the entire data set, defined and named. Writing of themes was an iterative and an integrated process throughout to aim for a comprehensive analysis. KG met with the youth peer evaluators, reviewed all coding and the identified themes to stimulate discussion and encourage reflexive acknowledgment of results and perspectives in the research process [37]. To aim for quality thematic analysis, the 15-point “checklist” for good thematic analysis was consulted (pg 96) [38].

## Results

**Fig. 1:**
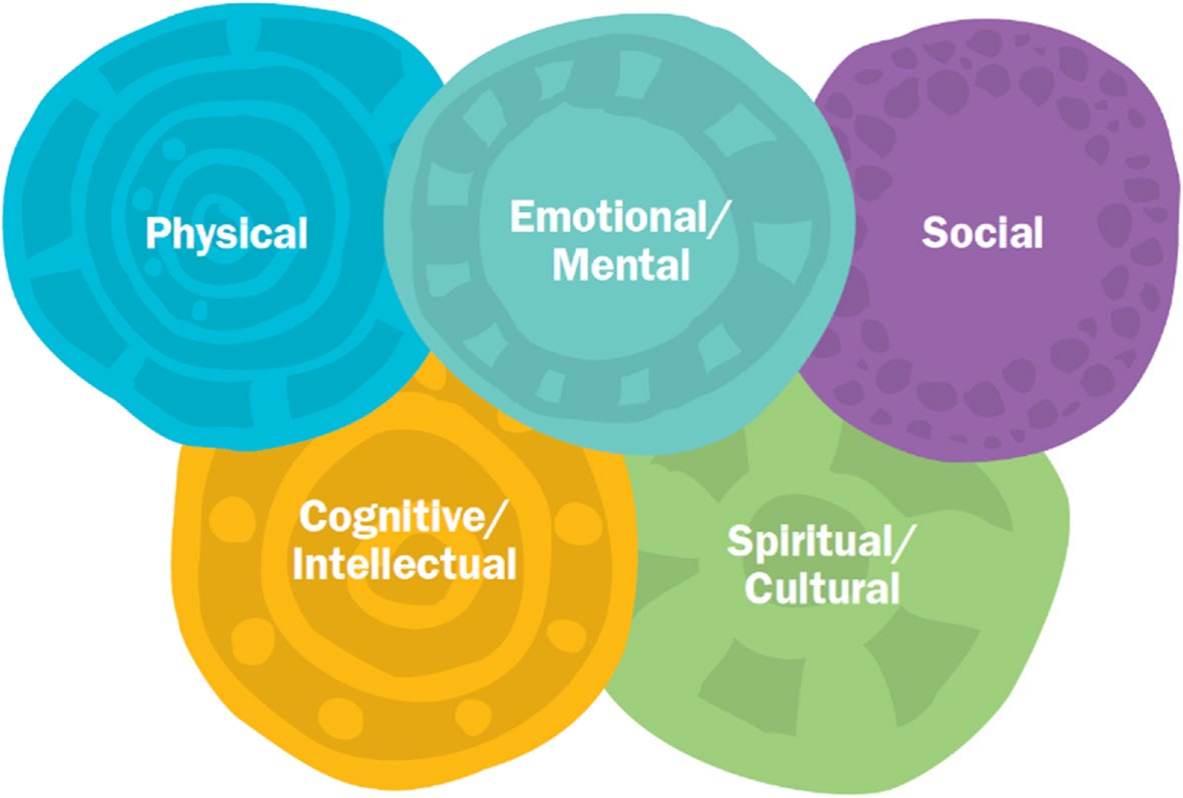
Wellness domains

**Fig. 2:**
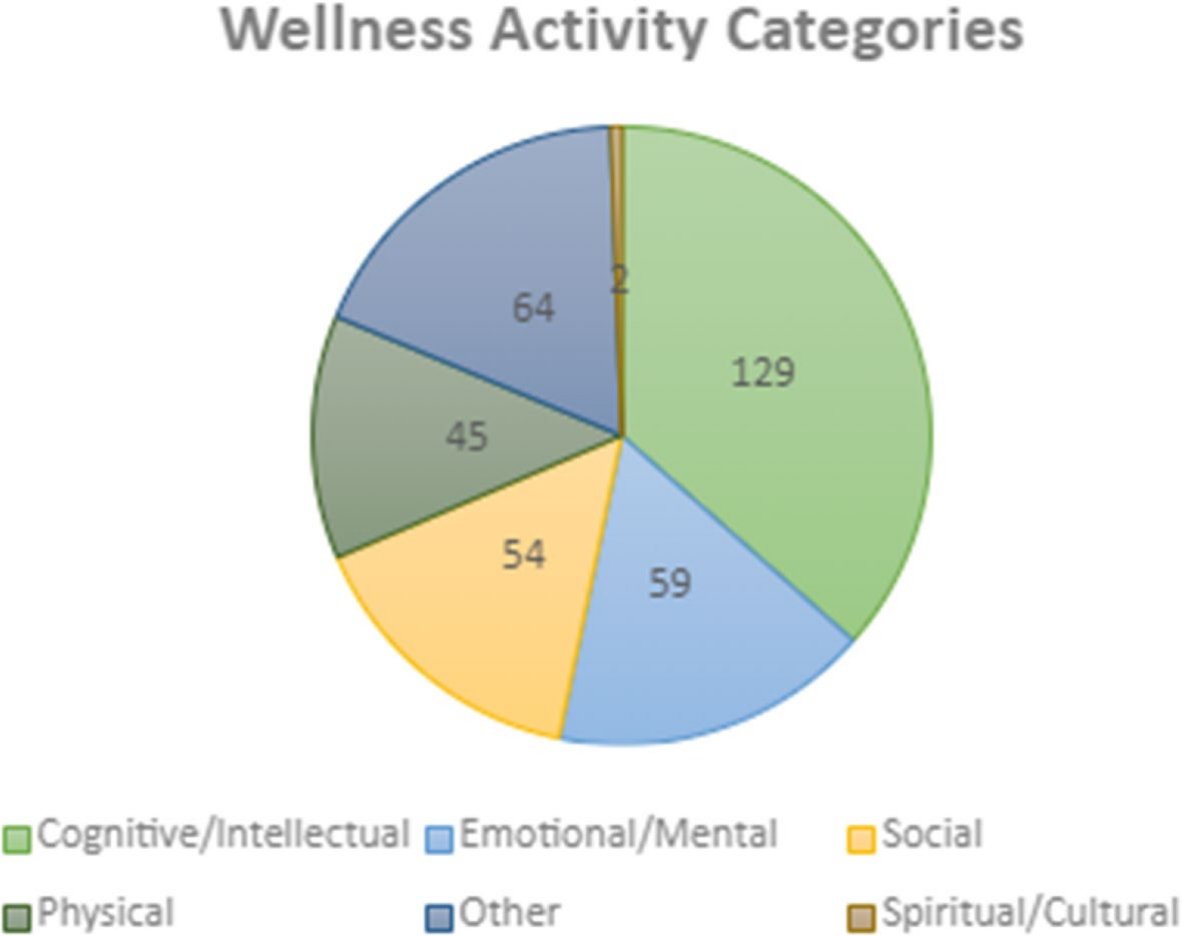
Wellness activities offered within each wellness domain

Results are based on ‘Toolbox’ data collected at Foundry centres over the two-year period after program launch (September 2019-September 2021). The results suggest a wide range of leisure activities offered within the Wellness Program across this IYS network. A total of 384 different leisure activities were offered within five wellness domains (Fig. 1). Table 2 provides an overview of each wellness domain, its definition, and examples of domain-promoting activities. Work by author BM (Fig. 2) provides a summary of the number of activities offered in each of the five wellness domains. The most commonly represented domain was cognitive/intellectual (*n* = 129; 36%), followed by emotional/mental (*n* = 59; 17%), social (*n* = 54; 15%), physical (*n* = 45; 13%) and spiritual (*n* = 2; 0.1%). There were 64 (18%) activities with acronyms and names that could not be classified and thus labelled as “other”. Examples include ‘KOB CMP’; ‘KVR RADAR’; ‘BLIP’; and ‘Drop-in’ (See Table 4 for a list of all activities). The Wellness Program offered a combination of individual, small or large group; community-based; in person and virtual activities. Examples of activities included Bob Ross Paint Nights, Book Clubs, Dungeons and Dragons, Hiking, Yoga, and Community Cooking. See Table 4 for a list of all names of activities in the Wellness Program offered and the corresponding wellness domain the activity targeted.

**Table 4:** Wellness program activities within wellness domains

**Wellness domain**	**Activities offered**
Cognitive/Intellectual	Activate: Tie Dye (Virtual)
	Activate: Tie Dying (In Person)
	Aromatherapeutic Crafts
	Art Social Group
	Art Therapy
	Art Therapy
	Art Therapy
	Art with Creative Life
	Arts and Crafts
	Beading Poppy
	Bob Ross Paint and Pizza
	Bob Ross Paint Night
	Book Club: Harry Potter ©
	Brownies on Thursday
	Candy Apples
	Cheap Eats Granville
	Cheap Eats St. Helen's
	Coffee & Creative Monday
	Community Meal Planning
	Cooking and Lifeskills
	Cooking Classes
	Craft Event
	Crayon Art
	Creative Journaling
	DIY Zen Garden
	Dungeons and Dragons
	Dungeons and Dragons
	Game nights
	GAME READY
	GAME READY
	Gamer's Unite
	Gamer's Unite
	Games Granville
	Gourd Painting
	Healthy Relationships Dialogue
	How to Survive a Pandemic: A Mindful and Artful Way
	Knitting
	Life Hacks
	Life Hacks
	Nintendo Switch Party
	Online: Rock Decorating
	Paint and Pizza
	Paint and Pizza
	Paper Quilling
	PCA Cooking
	Peer Support Baking
	Pottery Group
	Pottery Group
	Pottery
	Pottery
	Preserving Food
	Quilling
	Renfrew Cooking Group
	Renfrew Games Group
	Renfrew Gardening Group
	Renfrew Lifeskills
	Rock Painting
	Saint Helen's Clean Sweep
	Saint Helen's Cooking
	Saint Helen's Meal Planning
	Self-Esteem Workshop
	Self-Esteem
	Sketching in Nature
	Slime Making
	Sundaes
	TYES- D&D
	Virtual Bingo
	Virtual Games 0520
	Virtual Group: Drawing Tutorial
	Young Adult Game Night (17+)
	Youth D&D
	Community Cooking Group
	Cookies for Mental Health Week
	Kettle Breakfast
	Kettle Cooking
	Kettle Gardening Group
	Kettle Home Skills Drop-In
	Kettle Meal Planning
	Kettle on Burrard - Cheap Eats / Community Walk
	KOB Gardening Group
	KOB Gardening
	Make and Take Bannock Bites
	Maker Monday - Activity
	Mason Jar Crafts
	Mosaic Colouring
	Paint Like Bob Ross
	St. Helen's Cheap Eats
	Thursday Mosaic
	Thursday Painting
	Tie Dye on Thursday
	T-shirt Designs
	TYES - Arts & Crafts: Playdough
	TYES - Arts and Crafts - Coloring Page
	TYES - Arts and Crafts Bracelet Making
	TYES - Arts and Crafts Independence
	TYES - Arts and Crafts Rock Painting
	TYES - Arts and Crafts
	TYES - Bead Animal Making
	TYES - Collage Making
	TYES - Lava Lamps
	TYES - Mascaraed Mask
	TYES Arts and Crafts - Origami
	TYES Arts and Crafts - Rock Painting
	TYES Arts and Crafts - Tye Dye
	TYES ARTS AND CRAFTS GARDENING
	TYES Arts and Crafts
	TYES Arts and Crafts: Free for All Paint
	TYES Arts and Crafts: Light Sabers
	TYES Arts and Crafts-Bracelets
	TYES Arts and Crafts-Dream Catchers
	TYES Arts and Crafts-Firefly Jar
	TYES Arts and Crafts-Nutrition
	TYES Arts Crafts
	TYES- Healthy Boundary's
	TYES- Muffins
	TYES- Splatter Painting
	Tyes/arts and crafts Brownies
	Wellness Wednesday Goals
	Wellness Wednesdays - Internet Safety/Social Media
	Wellness Workshop Series - Belonging: Finding Our Kind
	Wellness Workshop Series - Food and You
	Wellness Workshop Series - Stuck on Sleep
	Wellness Workshop Series
Emotional/Mental	BCCYIC Week 2021: Movie Night
	Body Image 101
	CBT for Social Anxiety
	CBT Foundations for Anxiety/Depression
	CBT Foundations
	CBT Mood Management
	CBT Skills Group
	CBT Skills May 2021
	CBT
	Chill Out Friday - Disney's Mulan
	Chill Out Fridays - Disney Soul
	Cinema Therapy
	DBT Comp
	DBT Prep
	DBT Skills Group
	DBT Skills Training
	DBT Skills
	DBT Skills
	DIALECTICAL BEHAVIOUR THERAPY
	Eco Art Therapy
	EFFT Caregiver Group
	EFFT Caregiver Workshop
	Get Grounded
	Get Grounded
	Girls Group- Meditation
	ICY Art Group
	Mending Mindsets (Anxiety Group)
	Mind Me
	Mind Me
	Mindful Gardening Group
	Mindful
	MindShift Anxiety Group
	Positive Affirmations
	SP20 YMIND
	Spa Day
	Stress Management
	Therapeutic Arts
	Therapeutic Performance
	Wellness Wednesday Depression
	WW Anxiety
	YMIND
	Y-mind
	YMIND
	Youth Mind
	Youth Mindfulness
	Youth Mindfulness
Physical	Disc Golf
	Garden Group
	Healthy By Nature- Boundary Bay
	Healthy By Nature
	Healthy By Nature: Mike Lake
	Hiking Group - POWER TO BE
	Hixon Falls
	Indoor Rock Climbing
	KOB Coffee Walk
	LOUTET FARM GROUP
	Loutet Farm
	Lower Falls Hike
	Mindful Movement A
	Mindful Movement B
	Mindful Movement Yoga
	Mindful Movement
	Outdoor Sports
	Quarantine Fitness Crew
	Quarantine Fitness Crew
	Quarantine Fitness Group
	Rec Outing
	Renfrew Recreational Group
	Renfrew Walks
	RockWall Climbing Outing
	Self Defense with KB
	Skate Boarding
	Virtual Yoga
	Virtual Yoga
	Walking Group
	Water Balloon Fight
	Wild Play Adventures
	Workout for Wellness
	Workout in the Park
	Yoga Group
	Yoga Group
	Yoga
	Youth Group - Skating
	Youth Rec Night
	Zoomba Group
	Zoom-ba Group
Social	2SLGBTQAl+
	BBQ Night
	Caregiver Support Group
	Community HangOut
	Costume Community Clean Up
	D&D
	Foundry PG Youth Group
	Foundry Youth Group
	FPG Youth Group
	Girls Group
	Girls Group
	Girls Social Group
	LGBTQ2S+
	LGBTQ2S+
	Peer Group -
	PG Foundry Youth Group
	Pumpkin Carving Day
	Pumpkin Carving Event
	Queer Cafe© (FODxQC)
	Queer Cafe©
	Queer Cafe (18-24)
	Queer Cafe
	Queer Cafe
	STH Breakfast Group
	Talking Heads (Renfrew)
	Tuesday Chat on Zoom
	Youth Advisory Committee
	Youth Group
	Community CMP
	Fall 2021 UNYA 2S Group
	Friday 13th Youth Group
	FYG Movie Night
	Girls Group MC College
	Halloween Movie Night
	Hearing Voices Group
	Inner City Jamz
	Jurassic World Watch Party
	Monday Kettle Drop-In
	Social Summer Series
	Toast and Tea
	TransMission Peer Group
	TYES - Arts and Crafts - Karaoke
	TYES - Arts and Crafts Charades
	TYES - Arts and Crafts Movie The Simpsons
	TYES - Arts and Crafts Movie
	TYES - Arts and Crafts Movies
	TYES - Chill Out Friday Movie Night
	TYES Arts and Crafts - Bingo/Board Games
	Young & Recovering
	Young & Recovering
	Young and Recovering
	YOUnity Lounge
Spiritual/Cultural	Henna on a Thursday
	Thursday Beading

**Fig. 3:**
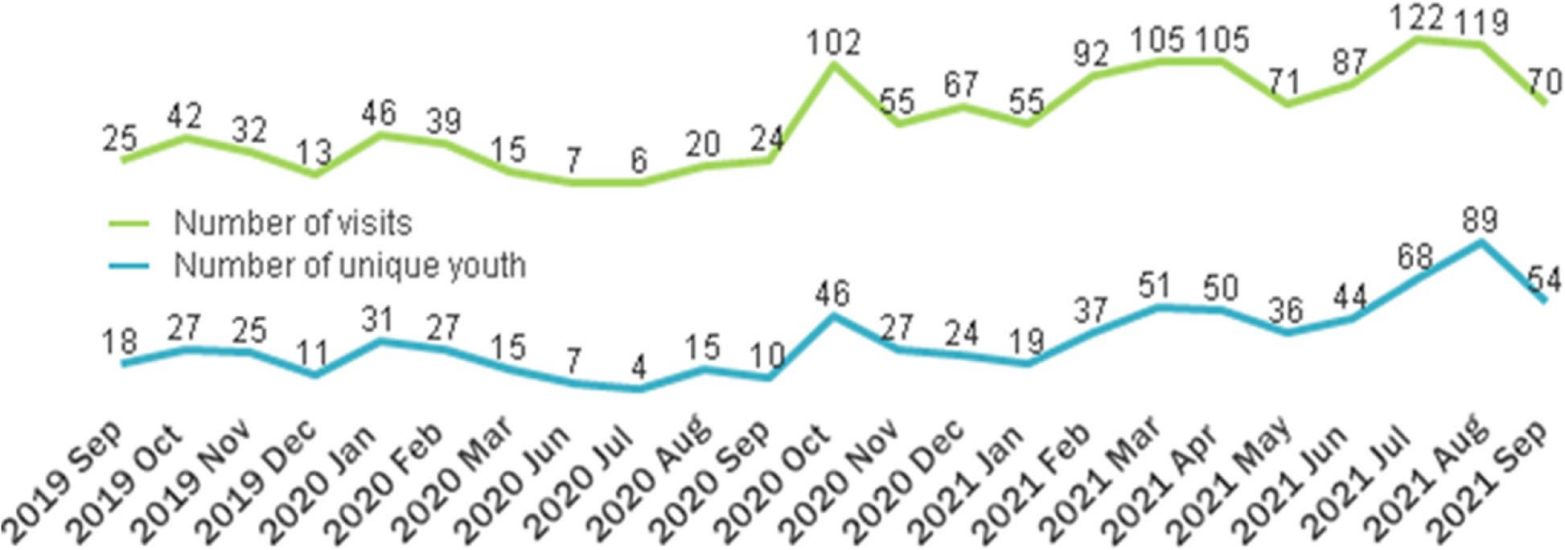
Number of unique youth and visits by month

**Fig. 4:**
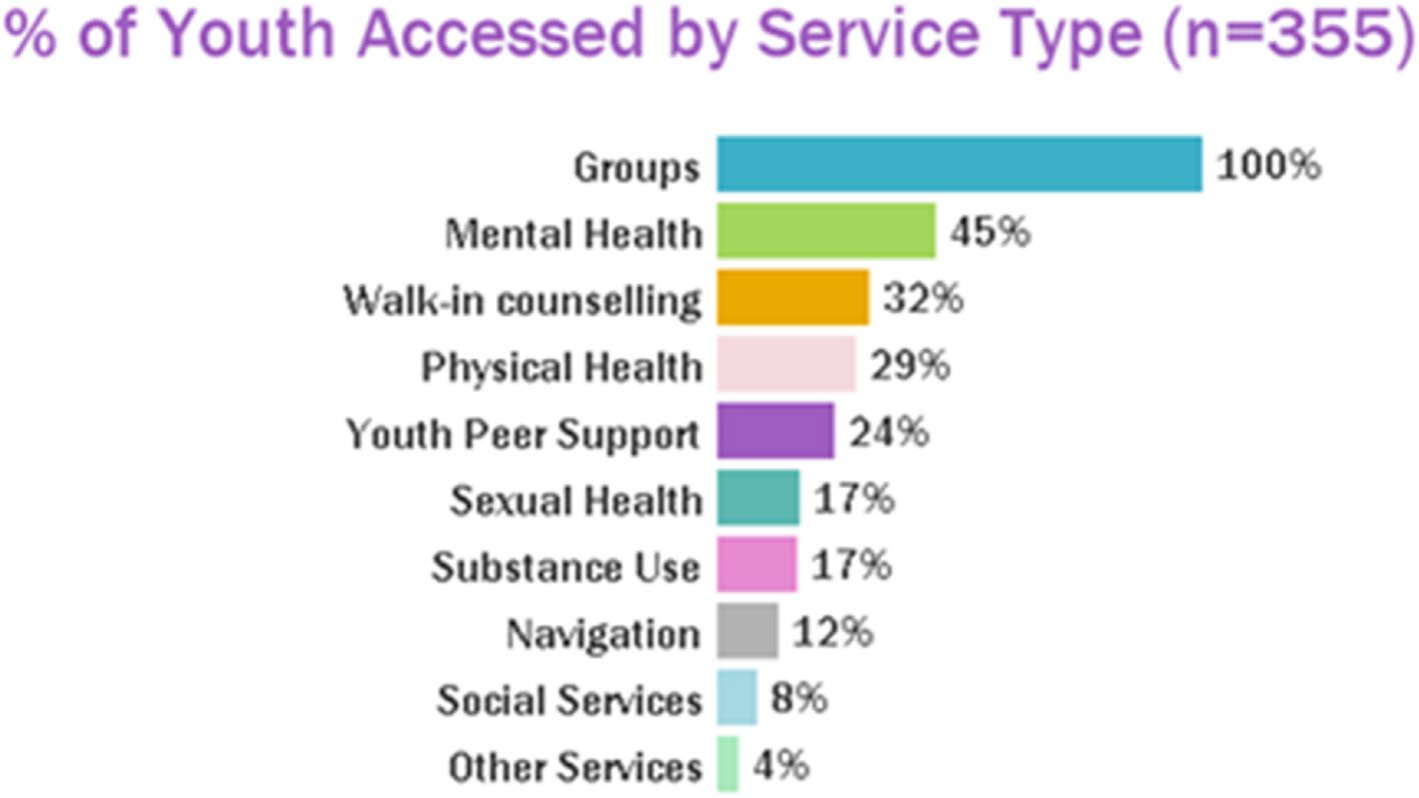
Summary of other foundry services accessed by youth participating in the wellness program

Three hundred and fifty-five unique youth accessed the Wellness Program, with 1319 unique visits. Figure 3 provides a summary of the number of unique youth and visits by month. The highest number of unique visits occurred in July 2021 (*n*=122; 9.2 %) and August 2021 (*n*=119; 9%), and no visits occurred in April and May 2020. The highest number of unique youth visited the program in August 2021 (*n*=89; 25%), and no youth visited in April and May 2020. Almost half (40%; *n*= 142) of youth identified that the Wellness Program was the first point of access to Foundry. In addition, 75% (*n*= 265) of youth accessed other programs at Foundry either before or after accessing the Wellness Program. The most commonly accessed services in addition to the Wellness Program were mental health (*n*=119; 45%), walk-in counselling (*n*=85; 32%), and physical health (*n*=76; 29%) (Fig. 4). When asked about how youth found out about Foundry (*n* = 175 responses), most identified a healthcare provider (*n*=36; 20.6%), school counselor/teacher (*n*=35; 20%), family member (*n*=32; 18.3%), friend (*n*=25; 14.3%), or a worker (*n*=20; 11.4%). When asked, “If the centre was unavailable, I would have gone to … ” (*n* = 173 responses), 56 (32.9%) said nowhere/I wouldn’t have gotten help, 38 (22%) said family members/friends, and 30 (17.3%) answered my healthcare provider.

The demographic profile of youth accessing programs is provided in Table 5. All demographic questions were optional, so the number of responses differ from the total number of youth accessing the program. The majority identified as young girls/women (*n*= 103; 58.2%), followed by gender diverse (*n*=40; 22.6%) or young boys/men (*n*=34; 19.2%). The mean age was 19 years, and most participants were between the ages of 19-24 years (*n*= 154; 43.6%). Youth identified as heterosexual (*n*= 69; 40%), bisexual (*n*= 41; 24%), gay or lesbian (*n*=15; 9%) or preferred not to answer (*n*=19; 11%). For ethnicity/cultural background, 104 (58.2%) young people were White, 35 (19.2%) Indigenous, 7 (3.9 %) Chinese, and 7 (3.9%) Filipino.

**Table 5:** Demographic characteristics of youth who accessed the wellness program

Gender identification, n (%)	Total responses *n* = 177
Young girl/woman	103 (58.2)
Gender diverse	40 (22.6)
Young boy/man	34 (19.2)
Age in years, n (%)	Total responses *n* = 353
<12 years	4 (1.1)
12-15 years	102 (28.9)
16-18 years	80 (27.7)
19-24 years	154 (43.6)
>24 years	13 (3.7)
Ethnic or cultural background, n (%)	Total responses *n* = 179
White	104 (58.2)
Additional options	41 (22.6)
Indigenous	35 (19.2)
Chinese	7 (3.9)
Filipino	7 (3.9)
Sexual Orientation, n (%)	Total responses *n* = 172
Heterosexual	69 (40)
Bisexual	41 (24)
Prefer not to answer	19 (11)
Gay or lesbian	15 (9)
Additional options	14 (8)
Questioning	10 (6)
Asexual	3 (2)

### Results of thematic analysis

A total of 9 young people participated in 2 focus groups. See Table 6 for a full break down of demographic information of participants. A total of 7 young people filled out the optional demographic survey. The average age of participants was 17 years, almost half (*n*=3) identified as female, and in regard to ethnic/cultural background almost half were Caucasian/white (*n*=3). Results specific to the evaluation objectives (described in Table 3) are presented here, further in-depth results of the thematic analysis will be presented elsewhere.

**Table 6:** Demographic characteristics of youth who participated in focus groups

**Demographic information**^a^	**Young people (*n*=7)**
Age (years)	Mean = 17
	Range = 15-23
Gender Identity	Female (*n*=3)
	Not sure/Questioning (*n*=2)
	Gender Fluid (*n*=1)
	Non-binary (*n*=1)
Ethnic/Cultural background	Cauasian/White (*n*=3)
	South Asian (*n*=2)
	Indigenous/Caucasian/Black (*n*=1)
	Indigenous/Caucasian (*n*=1)

^a^All demographic questions were optional, thus number of responses are different from the number of participants

#### Theme 1: Program successes

Young people explained that they appreciated and valued the social aspects of the program to connect with peers, and the facilitators, especially at a particularly isolating time during the global pandemic. Young people described the program facilitators with words such as “Welcoming; inclusive; accommodating; respectful; open to suggestions”. One young person explained the social aspect in further detail:

“ … people had the chance to sort of have banter and talk, if it's while cooking or painting and things like that which uh I think helped immensely when trying to make friends amid a pandemic... specifically for the online events, acquaintances and companionships were made”.

Another young person stated, “It helped with isolation and kept me from completely losing my marbles during quarantine”.

In addition to the social aspect, young people appreciated the opportunity to branch out and try new things. A young person explained:

“I found myself picking up different hobbies that I don't think I would've ever turned to if it wasn't for Foundry, such as painting, such as even making what was it, I think it was like mood boards. What I may have deemed as sort of like arts craftsy maybe childish, the Foundry embraced and really kind of captured it as an artistic and wellness activity”.

#### Theme 2. Desired program improvements

Young people talked about wanting more options for virtual or hybrid programming, specifically with accessibility interests in mind. Participants talked about how online events can reduce barriers to participation (such as transportation or social anxiety). One young person explained, “Having it virtually so we feel included and less isolated in person and virtually”. Another young person stated,

“ I mean it's nice through COVID it was definitely still through a screen, but even just baking cookies with everybody like on the phone kind of thing, it was nice, it was a good connection, it was good to get like hands on”.

Young people identified they wanted better communication about and within the programs. From program leaders, they wanted discussions around mental health and other health topics. One person stated “More in depth conversation would have helped me feel more connected”. Youth also discussed a lack of clarity around the program, and a lack of advertising about it. One person explained this, “My one criticism is there's like no marketing so like people don't know that Foundry holds socials and the only reason I know is ‘cause the youth worker in my school told me”. Another young person explained this communication breakdown as an accessibility issue:

“I do think it is a little bit hard to get involved in them … just because there tends to be tighter knit groups and also just like, not as much accessibility if you don't know what you're doing. I think it could be more clear, at least for my location where it is and what's happening and how to get into them”.

#### Theme 3. The ideal Wellness Program

Young people shared that their ideal program would be accessible, educational and fun, arts-based, social, and include a range of activities. One young person highlighted the need for social interactions and education, “I dislike the repetition of the same conversation topics that seems surface level. The topics don’t let us get to know each other very well”. Another young person summarized the need for a range of physical activities, “I would love to do activities like canoeing for example, because just being in the water, being out in nature, I have like a list written down of things I want to do after the pandemic is over. I would love to go bowling, go ice-skating, like using your body”.

## Discussion

To the best of our knowledge, this is the first study to describe development and phased implementation of leisure-based activities (known as the Wellness Program) across an IYS network. This study was done as part of a developmental evaluation [31, 32] to understand the complex system that is Foundry. At a provincial level, the BC Ministry of Mental Health and Addictions announced significant investment of government dollars for child and youth mental health support with prevention, early intervention, and wellness promotion being identified as key pillars [39]. This study helps to understand how an IYS such as Foundry can quickly adapt to the needs of youth and emerging circumstances (e.g., COVID) to co-design services that can be offered alongside traditional health services to address these key pillars. Promisingly, 355 unique youth accessed the Wellness Program over a two two-year period after program launch with 1319 unique visits. The highest number of visits occurred in July 2021 (*n*=122; 9.2 %) and August 2021 (*n*=119; 9%), with the greatest number of unique youth also visiting in August 2021 (*n*=89; 25%). No visits occurred in April and May 2020. This corresponds with the start of the COVID-19 pandemic lockdown, subsequent waves, and ease of restrictions in the province of BC. The province entered Step 3 of its Restart Plan July 1st, 2021, marked as a major milestone allowing indoor and outdoor group gatherings [40]. Almost half (40%) of youth identified that the Wellness Program was the first point of access to Foundry, indicating that these programs could be a gateway to other services and have the potential for early identification, intervention and treatment for other conditions. Youth commonly accessed mental health services (45%) in addition to the Wellness Program, indicating that young people may be participating in leisure activities as an adjunct service for their mental health. Perhaps this is due to program aspects (such as physical activity) having the potential to reduce anxiety and depression symptoms for young people [20] and potential to be a valuable mental health promotion tool [41]. Results from the focus groups with young people are important and being considered as the program continues to evolve and is scaled-up across the province at new centres. This includes the positive social aspect, a desire for hybrid in-person and online programming, improved communication in regard to what the program entails, and for Foundry to strive to offer an ideal program that is accessible, educational and fun, arts-based, social, and include a range of activities.

At a national level, youth mental health has been identified as a top priority for health care in Canada [42]. However, studies done during the pandemic have suggested mental health and substance use concerns for young Canadians [9, 43–45], identifying trans and gender diverse youth a particularly at-risk population during this time [45]. Hawke and colleagues [45] identified that trans and gender diverse youth are experiencing more mental health concerns, have fewer social supports, and have more unmet mental health and substance use service needs. In our study, 40 (22.6%) of youth identified as gender diverse (the second highest gender identity), and the most commonly accessed service in addition to the program was mental health services (45%). It is possible that these gender diverse youth accessed the program for reasons such as social supports, mental health concerns, and unmet service needs. A longitudinal study identified that mental health concerns of young people are evolving over time (in alignment with COVID-19 infection rates and potentially seasonal factors) [43]. The authors have recommended that it is essential to “engage directly with youth to cocreate pandemic response strategies and mental health service adaptations” (pg. 9) [43]. This study is an example of how the Wellness Program was co-created with diverse communities putting youth and families at the forefront and implemented as an adaptation to existing mental health services in one province. Co-designed leisure activities warrant consideration in other provinces within IYS and other health services as we continue to navigate the ongoing pandemic and future recovery.

At an international level, investment in and consideration of the long-term mental health needs of young people have been deemed essential [46]. Other work has been done in Sweden to advocate for the importance of leisure activities for young people for health promotion [47]; however, such programs are offered in youth centres separate from health and social services, placing a burden on youth and families to navigate different systems. In the United States of America, subsidized leisure activities for youth are offered within ‘afterschool programs’ and are embedded with other content (e.g. linked to education curriculum), not stand alone or considered part of health services [48]. A review done by Hetrick and colleagues [22] describes international IYS as offering services for mental health, substance use, physical health, and vocational and educational programs. The authors’ synthesis of IYS principles and characteristics included “Services may also provide recreational or arts activities and drop-in or hang-out space” (p. 56) [22]. Consistently, the engagement of young people in the design of services is an integral part of IYS [22, 24], and development and implementation of the Wellness Program was in response to an identified need of young people. This work provides a template to guide other international IYS and health service settings to develop, tailor, and implement customized programming.

### Strengths and limitations

This study provides novel insight into the development and implementation of unique leisure-based activities over a phased approach into IYS and offering alongside traditional health services as a means of health promotion. Although the sample size was small (*n*=9) the perspective of young people and their experience participating in the program as initial program evaluation has helped to understand the program and how it can be improved. The COVID-19 pandemic posed difficulties with navigating the offering, implementation, and tracking of programs. 'Toolbox' data did not capture youth participating virtually, thus the numbers reported in this study are likely an underrepresentation. Staff required 'Toolbox' training and had limited resources/capacity for data entry during the pandemic. The rapid development and expansion of Foundry centres pose challenges for research and program evaluation to keep up alongside. Each Foundry centre could develop its own unique program, which ultimately led to a wide variety of activities offered, and at times it was challenging to understand details (such as frequency, activities, group size).

### Future directions

As this work was part of developmental evaluation, multiple iterations over the course of the evaluation period are being done [31, 32]. Our team is working to analyze additional qualitative data (1-1 interviews with program staff) to better understand youth and program staff experiences for program improvement, and to establish how ‘Toolbox’ can be utilized for tracking virtual programs. Youth peer evaluators have presented the results from the focus groups with young people to FCO staff to initiate knowledge exchange. Our team is continuing to share results with staff offering programs (and developing new ones) as Foundry scales up across BC. Future research should explore the impact of the Wellness Program on short- and long-term health outcomes for young people and morbidity prevention considering pre-post and longitudinal designs. Future research could also explore cost-effectiveness of programs and the potential to reduce or mitigate wait times to other costly mental health services, as has been done in other settings such as a university campus and access to counselling services [49].

## Conclusions

This study provides novel insight into the development and implementation of leisure-based activities known as the Wellness Program into IYS which can be leveraged as a health promotion tool. This work provides a template for guidance that can be utilized within international IYS settings to develop, tailor, and implement similar programming suited to needs of young people, their families/caregivers, and communities. The initial reach of the Wellness Program over two years is promising, and it may be acting as a gateway for young people to access other health services. From initial program evaluation, young people enjoyed the social aspect with peers and facilitators, and identified important program improvements that are being considered as the program grows. Further work is needed to understand youth health outcomes and experiences associated with participation in such programs.  A multidisciplinary team is continuing to conduct evaluation of the Wellness Program using mixed-methods over multiple iterations as part of developmental evaluation.

## Supplementary Information

**Additional file 1.** 

## Data Availability

The dataset generated and analysed during the current study are not publicly available due to privacy reasons but are available from author SB on reasonable request.

## Abbreviations

MHSU: Mental health and substance use
IYS: Integrated youth services
BC: British Columbia
FCO: Foundry Central Office
FIPA: Freedom of Information Privacy Act
PIPA: Personal Information Privacy Act
