# Supplementary material for: Infusing wellness opportunities into integrated youth services
Source: BMC Psychiatry. 2023 Jun 5;23:403. doi: 10.1186/s12888-023-04809-6 (PMC10240455; doi:10.1186/s12888-023-04809-6)
Supplement: Supplementary file 1 — Additional file 1. [file 12888_2023_4809_MOESM1_ESM.docx]

**Supplementary Material**

1. Survey for Foundry Centres implemented in Phase 1: Community Asset Development

1. What does “wellness” mean to you?

2. What does “wellness” mean to the youth you serve?

3. What would an ideal wellness program look like for youth/young adults within your community?

4. What community resources/services do you currently utilize?

5. What are key areas of focus/priorities for youth in regard to wellness, leisure and community access?

6. What are the main barriers for youth in accessing these resources/ services?

7. What does your support system look like to facilitate youth voice, choice and leadership?

8. What makes your community unique with developing wellness and healthy lifestyle programs? Any unique features given geographical or cultural considerations?

9. What works well already with youth engagement and culturally responsive programming?

10. What would you like to see change with youth engagement and culturally responsive programming?

11. What are important considerations and values with diversity and inclusive practices? 12. Anything else that’s important to know before meeting with you and your community partners.

1. Focus Group Guide for Youth

| **Focus Group Guide Question** | **Optional Prompts** |
| --- | --- |
| 1. What did you like about the activities? What did you dislike?    1. What were your expectations going into the activity and did it meet/not meet/ exceed them? |  |
| 1. How was your experience with the activity leaders? | - Were they supportive, welcoming? How? - If not, why? |
| 1. How accessible was the program to participate in? 2. How could it have been more accessible? | - Considering different backgrounds, abilities and needs? Why or why not (Pros/cons)? - Did you feel welcome? |
| 1. What does wellness mean to you? |  |
| 1. How did participating in the wellness program impact your overall health? | - Mental health? Physical health? |
| 1. How has participating impacted your idea of self? | - Self-confidence, self-care, self-understanding |
| 1. After participating in activities, have you found similar activities in your community? How so? | - For example, did you continue hiking on your own outside of Foundry? |
| 1. This program was designed to focus on activities NOT dependent on use of a screen (for example the activity of painting). How did you feel about that? |  |
| 1. What was your experience to intentionally disconnect from screens?    1. How was your screen time impacted by COVID-19? | - How was your screen time impacted by participation in the activity? |
| 1. How did it feel to participate in this group with other folks?    1. How did you find participating in the program impacted your connection to others? | - Consider things like; being part of a group of friends; feeling less alone or isolated (especially during Covid); able to find companionship - What could have helped you feel more connected? |
| 1. Consider what program you participated in that involved physical activity or exercise. What do you like about physical activity and movement?   a. What do you dislike? |  |
| 1. If we were to create a physical activity program you could safely do during the pandemic with others, what activities would you be interested in? (consider covid- 19 pandemic precautions). Some examples include virtual group exercise, outdoor activities from a distance, time with animals, or a variety? | - Virtual group exercise class examples such as bodyweight exercises & strength training, Zumba, yoga, dance - Outdoor activities that can be done from a safe distance such as biking, walking, running, hiking, skiing/snowboarding - Time with animals, e.g. dog walking or horseback riding - A variety? |
| 1. If you could engage in any type of physical activity or exercise after/ outside of the pandemic what would it be? Consider group in-person classes, outdoor activities, team sports, time with animals, or a variety? | - Group in-person exercise class examples: strength training, zumba/dance, yoga, step classes - Outdoor activities such as biking, walking, running, hiking, skiing/snowboarding - Team sports such as soccer, football, baseball or something new such as gaelic football (a mix between rugby and soccer) - Time with animals - A variety of different things to determine what your preferences are? |
| 1. What do you know about the relationship between physical activity and mental health?    1. Would you like to know more about this? |  |
| 1. How did you hear about these activities?    1. What do you think are the most effective ways we could reach more youth to let them know about these programs? |  |
| 1. Is there anything else you would like to add or feel is important that we haven’t discussed yet? |  |
